# Identification of a combination of transcription factors that synergistically increases endothelial cell barrier resistance

**DOI:** 10.1038/s41598-020-60688-x

**Published:** 2020-03-03

**Authors:** Filip Roudnicky, Bo Kyoung Kim, Yanjun Lan, Roland Schmucki, Verena Küppers, Klaus Christensen, Martin Graf, Christoph Patsch, Mark Burcin, Claas Aiko Meyer, Peter D. Westenskow, Chad A. Cowan

**Affiliations:** 1 Pharmaceutical Research and Early Development, Roche Innovation Center Basel, F. Hoffmann-La Roche Ltd., Basel, CH-4070 Switzerland; 2 Pharmaceutical Research and Early Development, Ocular Technologies, I2O, Roche Innovation Center Basel, F. Hoffmann-La Roche Ltd., Basel, CH-4070 Switzerland; 3 Pharmaceutical Research and Early Development, Pharmaceutical Sciences, Roche Innovation Center Basel, F. Hoffmann-La Roche Ltd., Basel, CH-4070 Switzerland; 4 Department of Medicine, Division of Cardiology, Beth Israel Deaconess Medical Center (BIDMC), Harvard Medical School, Boston, Massachusetts, 02215 USA; 5 Department of Stem Cell and Regenerative Biology, Harvard University, Cambridge, Massachusetts 02138 USA; 6 Harvard Stem Cell Institute, Harvard University, Cambridge, Massachusetts 02138 USA

**Keywords:** Cell lineage, Stem-cell differentiation

## Abstract

Endothelial cells (ECs) display remarkable plasticity during development before becoming quiescent and functionally mature. EC maturation is directed by several known transcription factors (TFs), but the specific set of TFs responsible for promoting high-resistance barriers, such as the blood-brain barrier (BBB), have not yet been fully defined. Using expression mRNA data from published studies on *ex vivo* ECs from the central nervous system (CNS), we predicted TFs that induce high-resistance barrier properties of ECs as in the BBB. We used our previously established method to  generate ECs from human pluripotent stem cells (hPSCs), and then we overexpressed the candidate TFs in hPSC-ECs and measured barrier resistance and integrity using electric cell-substrate impedance sensing, trans-endothelial electrical resistance and FITC-dextran permeability assays. SOX18 and TAL1 were the strongest EC barrier-inducing TFs, upregulating Wnt-related signaling and EC junctional gene expression, respectively, and downregulating EC proliferation-related genes. These TFs were combined with SOX7 and ETS1 that together effectively induced EC barrier resistance, decreased paracellular transport and increased protein expression of tight junctions and induce mRNA expression of several genes involved in the formation of EC barrier and transport. Our data shows identification of a transcriptional network that controls barrier resistance in ECs. Collectively this data may lead to novel approaches for generation of *in vitro* models of the BBB.

## Introduction

Endothelial cells (ECs) from different organs display unique molecular^1^ and functional^2^ profiles. These organotypic profiles arise during endothelial cell development and are directed in part by signals from neighboring cells that activate TFs in ECs to activate or repress specific gene networks^3^. Organotypic differences are pronounced in ECs isolated from the central nervous system (CNS)^1,4–6^ that generate the blood-brain barrier (BBB), a highly selective and semipermeable barrier. Unique properties of the BBB include suppressed transcytosis, high tight junction and specialized transporter gene expression, and low immune cell adhesion gene expression^7^. Studies suggest^8–12^ that canonical Wnt, Hedgehog and retinoic acid pathways are involved in BBB development. However, other pathways are certainly involved, and the full set of TFs activated in ECs to generate the BBB has not been determined^13,14^. A more complete understanding of TF activation programs in CNS-derived ECs would greatly inform BBB biology.

To gain more insights into how and which TFs direct BBB formation, we perused the literature^4–6,8^ and identified several candidate TFs that could direct naïve ECs into BBB-like cells. Since stem-cell derived ECs are more amenable to gain-of-functional assays than primary cells^15^, we transduced combinations of candidate TFs in naïve human pluripotent stem cells derived ECs (hPSC-ECs)^16,17^ and measured gene expression, tightness and permeability of the cell-cell junctions. Using this approach, we identified a network of TFs (ETS1+SOX7+SOX18 and either TAL or LEF1) that significantly induced barrier resistance and induced expression of tight junction proteins and transporters characteristic of BBB ECs. These findings are important since improved BBB *in vitro* models may be useful for drug screening and functional testing.

## Results

To identify TFs that direct the differentiation of naïve hPSCs to BBB-like ECs, we first analyzed published gene profiling datasets from non- and CNS-derived murine ECs (GSE35802^8^, GSE48209^5^, GSE56777^4^, and GSE47067^1,6^). We entered the datasets with the largest number of tissues into RankProdit, a tool that compares multi-array data based on rank-model^18^ (Supplementary Dataset 1). Human and murine TFs were subsequently filtered from the dataset according to RIKEN TFs database^19^ (Supplementary Dataset 2). Studies based on similar tissue comparisons were used for validation (Supplementary Dataset 3)^4–6,8^. Finally, we excluded TFs with RankProdit fold-change values of <1.5 (based on GSE47067^1,6^; Supplementary Fig. 1). We then included some TFs based on literature compelling evidence (summarized in Supplementary Dataset 4).

**Figure 1:**
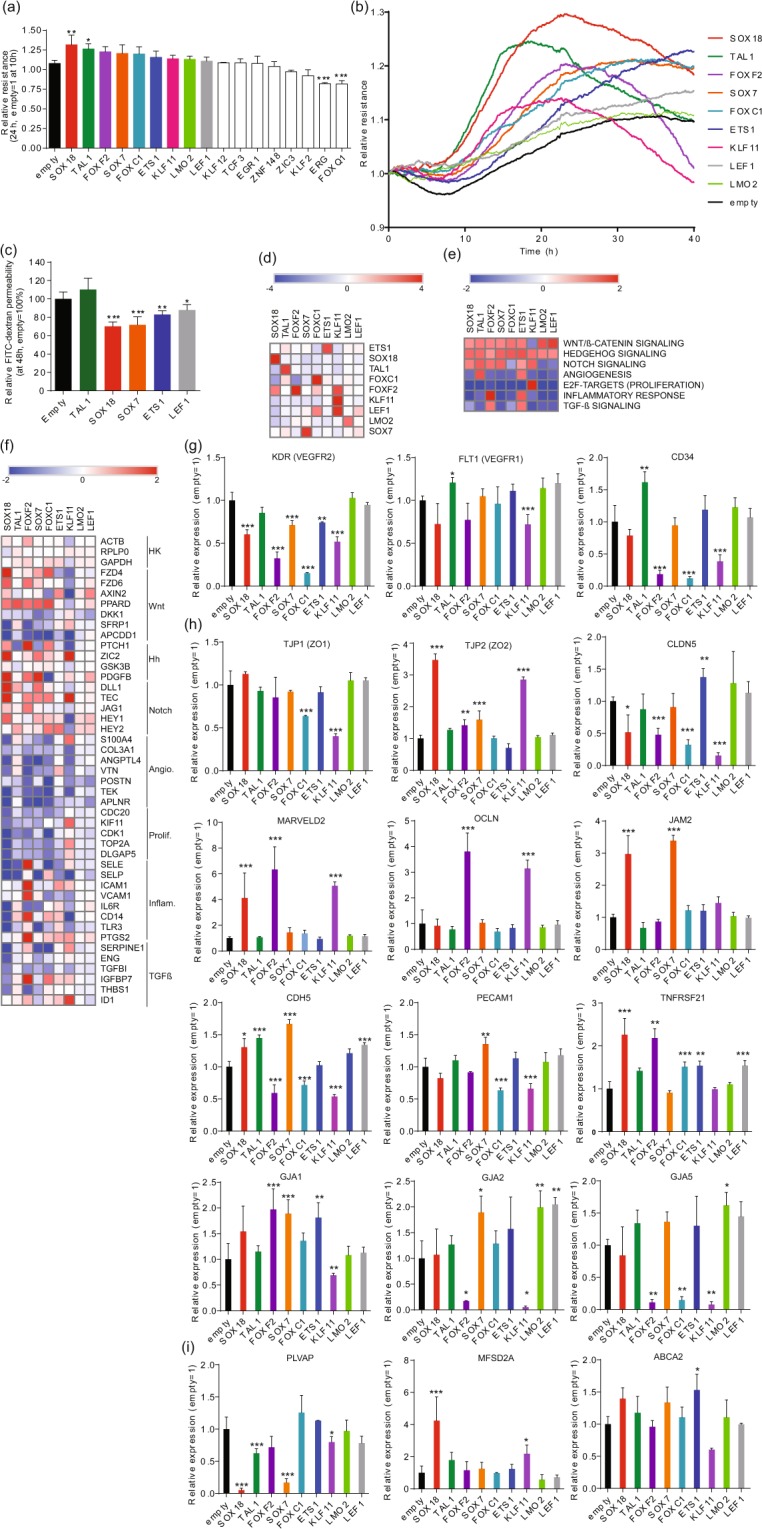
Identification of transcription factors that promote endothelial barrier resistance. (**a**) Mean relative barrier resistance at 24 h (80 MOI adenovirus) post-stabilization of the resistance measurement (measured post-stabilization of resistance measurement, which happens at 10 h after transduction); averages are from 3 independent experiments measured using ECIS. (**b**) Real time ECIS measurements for each of the TFs that demonstrated a positive effect on barrier resistance at 24 h in three independent experiment (measured post-stabilization of resistance measurement, which happens at 10 h after transduction). The lines denote the mean resistance. (**c**) FITC-dextran permeability assay at 48 h post-transduction; averages from 3 independent experiments. (**d**) Heatmap of log_2_ fold-change expression of TFs (rows) as measured by RNA-seq at 48 h post-transduction (80 MOI adenovirus) versus adenovirus empty vector control (columns). (**e**) Heatmap of normalized enriched scores (NES) generated by Gene Set Enrichment Analysis (GSEA) using the hallmark gene set at the MsigDB focusing on pathways known to be involved in EC barrier formation. (**f**) Heatmap of log_2_ fold-change expression of genes annotated to pathways analyzed by GSEA. (**g**) Relative mRNA expression of EC marker genes, (**h**) EC paracellular barrier genes, and (**i**) Transcellular EC transporters as compared to empty vector adenovirus control. Columns represent mean ± SD. *P or FDR < 0.05, **P or FDR < 0.01, ***P or FDR < 0.001. All experiments were performed in triplicates.

Using the criteria described above, we identified 17 TF candidates, and tested them using gain-of-function assays in hPSC-ECs (via adenovirus transduction; 80 MOI). The effects were measured using Electric cell-substrate impedance sensing (ECIS) after resistance values are known to stabilize (10 h post-transduction). Some TFs (TAL1 and SOX18) induced significantly enhanced barrier properties, and a positive trend was seen when seven others were transduced (colored bars) (Fig. 1a). Real-time ECIS data showed that TAL1 induced more rapid and dramatic effects than SOX18 (Fig. 1b). ETS1 also induced high resistance, albeit more slowly (Fig. 1b). Permeability assays using FITC-dextran were also employed; permeability was reduced in cells transduced with SOX18, SOX7, ETS1 and LEF1 48 h post-transduction (Fig. 1c). Somewhat paradoxically, no effect was observed in TAL1 transduced cells (Fig. 1c). However, ECIS effects due to TAL1 overexpression were also abolished at 48 h due to rapid activity of TAL1 (Fig. 1b).

We then set out to determine the molecular basis of the candidate TF activities using gene profiling. Gene profiling analyzes (RNA-seq; 48 h post-transduction) confirmed that transduced TFs were significantly upregulated (Fig. 1d, Supplementary Dataset 5). For this, GSEA^20^ data were analyzed using the Molecular Signatures Database (MSigDB) hallmark gene set^21^ focusing on pathways relevant to EC barrier integrity (Fig. 1e, f, Supplementary Dataset 5). Data showed that Hedgehog-related and canonical Wnt-related signaling family members were upregulated by all TFs tested except by KLF11, and ETS1 (and FOXF2 exerted the broadest effects). ETS1 and TAL1 activated angiogenesis-related genes, and KLF11 uniquely activated proliferation-related genes. The effects of SOX18 and SOX7 transduction largely overlapped (converging on canonical Wnt, Hedgehog and Notch pathways). FOXF2, FOXC1 and KLF11 reduced expression of classic EC markers VEGFR2, VEGFR1 and CD34 (Fig. 1g, Supplementary Dataset 5) and were therefore removed from future considerations.

We then set out to determine which of our candidate TFs altered expression of adhesion and transporter genes critical for the BBB: paracellular barrier^7,22^; tight junctions [TJP1 (ZO1), TJP2 (ZO2), CLDN5, MARVELD2, OCLN]; adhesion molecules [JAM2, CDH5, PECAM1]; gap junctions [GJA1, GJA2 and GJA5]; TNFRSF21^8^; and transcellular transporters [PLVAP, MFSD2A, ABCA2; (Supplementary Dataset 5; Fig. 1h, i)]. SOX18 and SOX7 activated the most paracellular transport genes [TJP2 (ZO2), MARVELD2, JAM2, CDH5, PECAM1, TNFRSF21], while TAL1 and LEF1 induced VE-Cadherin (CDH5^22^) (Fig. 1h). Of all the TFs tested, only ETS1 induced CLDN5 (and GJA1) (Fig. 1h). Both KLF11 and FOXF2 induced MARVELD2 and OCLN, but repressed CLDN5 and adherens and gap junction molecules (Fig. 1h). SOX18, SOX7 and TAL1 strongly downregulated PLVAP^23,24^, and MFSD2A^4^ was strongly induced by SOX18. ETS1 induced expression of ABCA2^25^ (Fig. 1i). We also checked expression of several other ECs transporters that were previously identified to be present on ECs of BBB (Supplementary Fig. 2) and observed strong upregulation of ABCG2 (BCRP^26^), SLC2A1 (GLUT1^27^), SLC1A1^28^, MAOA^29^, CAV2^30^, LEPR^31^ and ABCB1 (MDR1^32^) by FOXF2 (Supplementary Fig. 2). KLF11 overexpression induced strong upregulation of ABCB1 (MDR1), SLC1A1, TFRC^33^ and SLC38A5^34^ (Supplementary Fig. 2).

**Figure 2:**
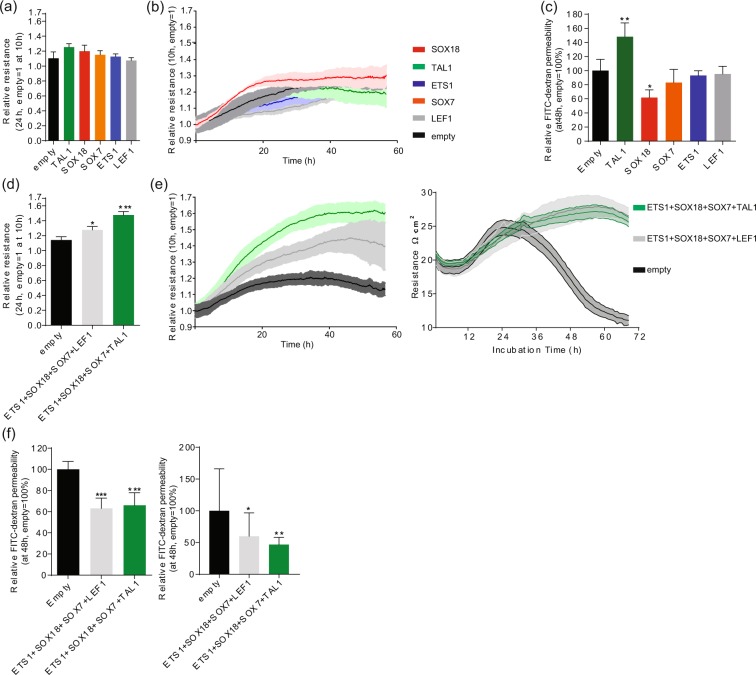
Transcription factor combinations synergistically induce endothelial cell barrier resistance. (**a**) Mean relative barrier resistance at 24 h (20 MOI adenovirus), post-stabilization of the resistance measurement (measured post-stabilization of resistance measurement, which happens at 10 h after transduction); averages are from 3 independent experiments measured using ECIS. (**b**) ECIS real-time EC barrier resistance after adenovirus transduction (each at 20 MOI). The lines denote the mean resistance and shading corresponds to standard deviation. (**c**) FITC-dextran permeability assay at 48 h post-transduction; averages are from 3 independent experiments. (**d**) Mean relative barrier resistance at 24 h of a combination of 4 adenoviruses each (each at 20 MOI), post-stabilization of the resistance measurement (measured post-stabilization of resistance measurement, which happens at 10 h after transduction); averages are from 3 independent experiments. (**e**) Real-time measurements of EC barrier resistance by ECIS (left) and TEER (right) for the combinations of TFs (each at 20 MOI). The lines denote the mean resistance and shading corresponds to standard deviation. (**f**) FITC-dextran permeability assay at 48 h post-transduction with 40 kDa (left) and 4 kDa (right); averages are from 3 independent experiments. Columns are mean ± SD. All transductions were performed as 3 independent experiments with at least 3 technical replicates. *P < 0.05, **P < 0.01, ***P < 0.001.

**Figure 3:**
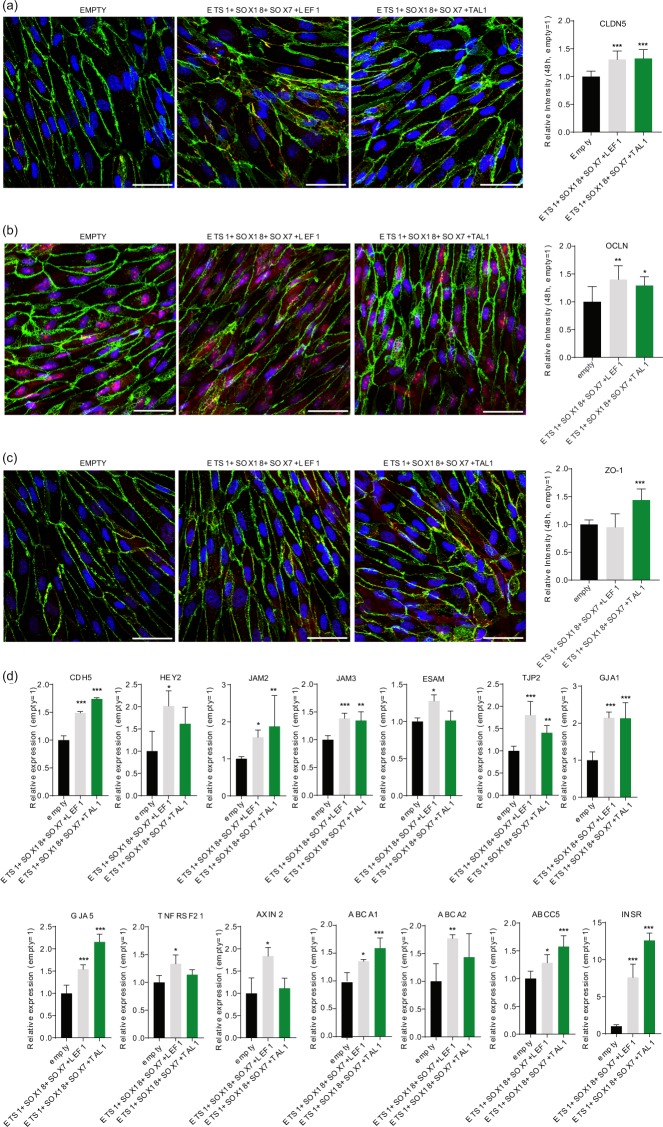
Transcription factor combinations synergistically induce endothelial cell junction proteins expression and mRNA expression of EC barrier relevant genes. Immunocytochemistry with confocal imaging and ImageJ analysis of (**a**) CLDN5, (**b**) OCLN and (**c**) ZO1 (all in red) after 48 h treatment with combination of TFs. As EC barrier marker, VE-Cadherin was stained in green and nucleus with DAPI in blue. Scale bar represents 50 µm. (**d**) Relative mRNA expression of genes involved in barrier junction and transport across EC barrier after treatment with combination of TFs for 48 h. Columns are mean ± SD. All transduction were performed as 3 independent experiments. *P < 0.05, **P < 0.01, ***P < 0.001.

Since TFs rarely operate independently^35^, we next forced expression of quadruple combinations of the promising candidate TFs identified in our functional barrier assays (Fig. 1a–c) but at lower MOI (20 each; 80 MOI total) in hPSC-ECs. First, ECs were transduced with individual TFs with 20 MOI, and no significant resistance increase was observed 24 h post-stabilization resistance (Fig. 2a), except for SOX18 that induced a modest increase (Fig. 2b). Only SOX18 decreased barrier permeability in FITC-dextran permeability assay (Fig. 2c). For combinatorial gain of function assays, we chose TFs that induced more resistant barrier in functional assays (Fig. 1b; SOX18, TAL1) with TFs that induced genes involved in barrier formation (Fig. 1h; ETS1, SOX7). Gene profiling assays suggested that the combinations of ETS1, SOX7 and SOX18 induced the largest number of barrier-related genes (Fig. 1h, Supplementary Fig. 2). Addition of either LEF1 or TAL1 to that TF combination led to an additional significant increase in barrier resistance (Fig. 2d,e). The highest induction was 1.7-fold (combination with TAL1), which is more than the additive effect of the 4 factors (at 20 MOI) and also higher than overexpression of the single most efficient TF when transduced at 80 MOI (Fig. 2b). There was a significant reduction in FITC-dextran permeability (both 4 and 40 kDa) for both combinations of TFs (Fig. 2f). Next, we focused on expression of the three most important tight junction molecules for EC barrier integrity CLDN5^36^ (Fig. 3a), OCLN^37^ (Fig. 3b) and ZO1 (TJP1^38^, Fig. 3c) by immunocytochemistry and we observed significant induction of protein expression of CLDN5 and OCLN with both TF combination and induction of ZO1 by overexpression of combination that included TAL1. To understand more broadly the effect on barrier integrity by the TF combinations overexpression we have performed global expression profiling by RNA-seq (48 h post-transduction, Supplementary Dataset 6) and observed induction of a number of genes involved in EC barrier integrity (Fig. 3d; CDH5, HEY2, JAM2, JAM3, ESAM, TJP2, GJA1, GJA5, TNFRSF21 and AXIN2) and transporters over EC barrier (Fig. 3d; ABCA1, ABCA2, ABCC5, INSR).

## Discussion

The molecular profiles of CNS-derived ECs have been well characterized^1,4–6^; what sets our study apart from the others is that we characterized the effects of candidate TFs using combinatorial gain-of-function assays. TFs are activated in key temporal sequences to regulate gene expression and to control key steps in development^39^. However, the transcriptional program required to generate a mature BBB is incompletely understood^13,14^. We selected candidate TFs responsible for BBB maturation with this criteria: (1) by comparing organotypic differences between non- and CNS-derived ECs; (2) based on compelling literature evidence (Supplementary Dataset 4). We overexpressed the candidate TFs in naïve (immature) human pluripotent stem cell-derived ECs (hPSC-ECs)^16,40^. We measured the effects using impedance and FITC-dextran permeability (paracellular permeability) assays (but did not explore the transcellular permeability).

From the gene expression comparisons, we selected these candidates: LEF1, FOXF2, FOXF1, and ZIC3. In our hands, LEF1, which may interact with β-catenin to regulate gene expression of ECs in BBB^41^, significantly increased barrier resistance post transduction. FOXF2 was shown to be involved in EC BBB development and maintenance^42^, but in our study, FOXF2 overexpression induced expression of several important EC tight junctions, but reduced CLDN5 (Fig. 1h). FOXF2 overexpression had only a modest effect in increasing resistance of EC barrier (Fig. 1a), and therefore was not used in subsequent combinations. FOXF2 may act synergistically with FOXF1^43^, which regulates EC barrier of lung ECs^44^. Therefore, it would be interesting in further studies to determine if FOXF2 and FOXF1 simultaneous overexpression would increase EC resistance. Finally, ZIC3 may be acting uniquely in CNS ECs to regulate the epigenetic status of CNS ECs (methylation and chromatin structure). qRT-PCR analyses showed that ZIC3 induced PECAM1 expression (data not shown) but did change the barrier properties of ZIC3-transfected ECs.

We continued to add candidates to our final combination of TFs based on the observations summarized below. ETS1 was included in subsequent combinations because it slowly improved barrier integrity post-transduction (Fig. 1b), and it is the only candidate TF that activated CLDN5 (Fig. 1h). In our hands, both SOX7 and SOX18 induced higher barrier resistance in accordance with another study showing that SOX7, SOX17 (not tested in our study) and SOX18 contribute to murine blood retinal barrier generation^45^ (Fig. 1b). TAL1 rapidly strengthened the barrier properties of ECs and TAL1 is reported to be involved in EC specification^46^. LYL1, a close homolog in TAL1, may be to be critical for generating the barrier in lung ECs.

Combinatorial interaction among TFs is critical for the differentiation of various cell types^35,47^, and here we determined that the combination of SOX18, SOX7, ETS1 and LEF1 (or TAL1) synergistically induces EC barrier resistance in hPSC-ECs. The combination of these factors was able to induce significantly protein expression of important tight junction proteins (Claudin5, Occludin, ZO1) and induced mRNA of several other important molecules involved in EC barrier integrity and transport across the barrier. The characterized combination of TFs could be used by researchers to generate optimized BBB models. Our gene-profiling results, collected after overexpressing nine TFs TAL1, FOXC1, KLF11, FOXF2, SOX18, LMO2, LEF1, SOX7 and ETS1, or a combinations thereof, could also serve as a comprehensive resource for vascular biologists.

## Methods

### Human PSC culture and differentiation

The hPSC line SA001^48^ (Cellartis AB) was differentiated as described^16,17^ with the following modifications. Expansion medium consisting of StemPro with 50 ng/mL of VEGFA was used for the first cell division, after which cells were cultured using VascuLife VEGF Endothelial Medium Complete Kit (LifeLine Cell Technology). The final composition of supplements added to the media was as follows: 10% FBS, 4 mM L-glutamine, 0.75 U/mL heparin sulfate, 5 ng/mL FGF-2, 5 ng/mL EGF, 5 ng/mL VEGFA, 15 ng/mL IGF1, 1 µg/mL hydrocortizone hemisuccinate, and 50 µg/mL Ascorbic acid. SB431542 (10 µM) was supplemented to the media.

### Adenovirus production

We tested several different promoters (UbiC, E1Fα and CMV) with GFP expression and several different multiplicities of infection (MOI) for introducing adenovirus (data not shown) and selected the UbiC promoter. Experiments with adenovirus expressing GFP with 20 MOI and 80 MOI were performed to identify MOI that would transduce all cells and show moderate expression of (20 MOI) or high expression (80 MOI), data not shown. The final construct had the UbiC promoter followed by the ORF and then the 3′UTR of GFP with a polyadenylation sequence. For all the TFs ORFs the consensus CDS (CCDS) or the most abundant expressed form of transcript in hPSC-ECs was cloned. Bacterial artificial chromosomes (BAC) were generated by transformation and recombination in *E. coli* DH10B carrying SIR-BAC-Ad5 encoding an E1- and E3- deficient adenovirus genome. Recombinant adenovirus particles were generated by transfecting the linearized construct into HEK293 cells and purifying the replication-competent adenoviruses using AdenoONE Purification Kit (Sirion Biotech). Adenovirus production was done by Sirion Biotech.

### Electric cell-substrate impedance sensing (ECIS)

96-well plates (Applied Biophysics) were coated with 100 μL of fibronectin (25 μg/mL), which was then replaced with complete media and electrodes were allowed to stabilize. hPSC-ECs were seeded at 10,000 cells/well and grown for 2 days to reach full confluence before transduction with adenovirus overexpressing TFs. ECIS was measured in real time using the ECIS® Z-theta system^49^ (Applied Biophysics) at 250 Hz^50^.

### Transendothelial electrical resistance (TEER)

The TEER values of hPSC-ECs were measured using the cellZscope (nanoAnalytics, Münster, Germany). Cells were prepared on HTS Transwell® -24 Well format (Corning; 3378) at 0.4 × 10^6^ cells/cm^2^ and let them grow for two days in order to form a monolayer. Afterwards SB431542 10 µM or RepSox 10 µM or DMSO were added on the apical chamber. The TEER values were recorded in real-time every 30 minutes automatically in triplicates.

### FITC-dextran permeability assay

hPSC-ECs were seeded in fibronectin-coated Transwell® 96-well plates (pore size: 0.4 µm, Corning) in complete media. EC media was added to both the bottom chamber (235 μL) and the top chamber (75 µL). Cells were incubated for 2 days to allow attachment and to generate a confluent monolayer before transduction with adenovirus overexpressing TFs in the upper chamber. Analysis was carried out 2 days later by adding 10 μL of 4 kDa or 40 kDa FITC-dextran to the top chamber and then by incubating for 30 min at 37 °C under 5% CO_2_. After 30 min, top wells were removed and the bottom plate, containing media and leaked FITC-dextran, was measured in a fluorescent reader (Molecular Devices, excitation 485 nm, emission 535 nm).

### Cell lysis and RNA isolation

Cultured ECs were lysed using 350 μL RLT lysis buffer (Qiagen) with 1% β-mercaptoethanol and subsequently vortexed for 1 min at room temperature before snap freezing. RNA was isolated from cell lysates using an automated Maxwell Total RNA purification kit (Promega) with DNAse I digestion.

### Immunocytochemistry

The hPSC-ECs were plated on fibronectin (Corning; 356008)-coated glass at 63,000 cells/cm^2^ in 24 well culture plate. When the cells formed monolayers, the cells were treated with 80 MOI empty vector, TAL1 TFs combination (ETS1+SOX18+SOX7+TAL1) and LEF1 TFs combination (ETS1+SOX18+SOX7+LEF1) at 20 MOI respectively. After 48 hours incubation, the hPSC-ECs were fixed in 4% PFA for 20 minutes at room temperature after PBS washing (including Mg^2+^/Ca^2+^). Then, cover slips were washed two times with PBS again, then blocked in SuperBlock™ (Thermo; 37515) with 0.3% TritonX for three hours at 4 °C on the shaker. Cells on the cover slip were stained with the primary antibody Human VE-Cadherin (R&D systems; AF938; 1:100), Claudin5 Polyclonal Antibody (Invitrogen; 341600; 1:100), ZO1 Polyclonal (Invitrogen; 402200; 1:100) and Occludin Polyclonal (Invitrogen; 711500; 1:100) overnight at 4 °C in 1/10 diluted SuperBlock™ in PBS^−/−^ solution with 0.3% TritonX. A day after, cells were washed three times for 10 minutes using PBS^−/−^ with 0.05% TWEEN 20, then incubated them with secondary antibodies [Alexa Fluor™ 546 donkey anti-Goat (Invitrogen; A11056; 1:200); Alexa Fluor™ 647 donkey anti-Rabbit (Invitrogen; A31573; 1:200)] for three hours at 4 °C on the shaker. After three times of washing with PBS^−/−^, Coverslips were mounted on glass-slide with Fluorescence Mounting Medium (DAKO; S3023) including DAPI.

### Imaging and quantification

Images were prepared using ZEISS LSM 710 confocal microscope with 40x objective (Carl Zeiss Microscopy GmbH). The image data was reconstructed into a single image for each Z-stack using maximum intensity projection function in ImageJ. The intensity of entire area of fluorescence images was taken into account for analysis.

### RNA-sequencing and analysis

Total RNA was subjected to oligo (dT) capture and enrichment, and the resulting mRNA fraction was used to construct cDNA libraries. Sequencing was performed on an Illumina HiSeq platform using the standard protocol (TruSeq Stranded Total RNA Library, Illumina) that generates approximately 30 million reads of 50 base pairs per sample. Reads were mapped to the human genome (either hg19 or hg38/Refseq) using either STAR or SAMtools^51,52^ and counting was performed using the union mode of HtSeq.^53^. Differential expression was performed using DESeq. 2 with the FDR correction^52^ and read counts for RPKM were normalized by sequencing library size and gene length according to the reference^54^. Gene set enrichment analysis (GSEA)^20^ was performed using the hallmark gene set at the MsigDb database^21^ with weighted analysis to sort the gene list based on fold-change from upregulated to downregulated genes using default conditions and ignoring gene sets smaller than 15 or larger than 500 genes. The generated RNA-seq files have been deposited to GEO (GSE142132).

### Statistical analysis

Prism 7 (GraphPad) was used for statistical analysis, which was an unpaired, two-tailed Student’s *t*-test unless mentioned otherwise (RNA-seq analysis). For all bar graphs, data are represented as mean ± SD. *P* values <0.05 were considered significant.

## Supplementary information

Supplememtary Information.

Supplementary Table 1.

Supplementary Table 2.

Supplementary Table 3.

Supplementary Table 4.

Supplementary Table 5.

Supplementary Table 6.

## Data Availability

RNA-seq files have been deposited to GEO (GSE142132).
