# Supplementary material for: Identification of a combination of transcription factors that synergistically increases endothelial cell barrier resistance
Source: Sci Rep. 2020 Mar 3;10:3886. doi: 10.1038/s41598-020-60688-x (PMC7054428; doi:10.1038/s41598-020-60688-x)

Supplementary Figure and Supplementary Dataset legends:

Identification of a combination of transcription factors that synergistically increases endothelial cell barrier resistance

Filip Roudnicky[1], Bo Kyoung Kim[2], Yanjun Lan[1], Roland Schmucki[3], Verena Küppers[2], Klaus Christensen[1], Martin Graf[1], Christoph Patsch[1], Mark Burcin[1], Claas Aiko Meyer[1#], Peter D. Westenskow[2*#], Chad A Cowan[4,5,6*#].

*Corresponding authors: ccowan@bidmc.harvard.edu, peter.westenskow@roche.com

Supplementary Material and methods

Analysis of published gene expression datasets. Raw Affymetrix expression files were downloaded from GEO (GSE35802 [1], GSE48209 [2], GSE56777 [3], GSE47067 [4, 5]) and analyzed using the Partek Genomic Suite. Probeset data were normalized by making pre-background adjustments for GC correction and probe sequence bias, and then correcting for RMA background and carrying out quantile normalization. Normalized probe set data were $\log_2$ transformed and summarized using the median polish method. Differential expression was determined using an ANOVA model. Data from a study by Nolan *et al.* [4], where brain ECs were compared to several other tissues, were analyzed using RankProdIt [6], a rank based meta-analysis tool that we used to generate a consensus brain EC gene signature. Additionally, a literature search was conducted to find evidence of TFs that are associated with EC barrier resistance formation [7-15] or induction of molecules involved in barrier formation (e.g. tight junctions [7-9, 12, 16-27]).

Supplementary Figure and Supplementary Dataset legends:

Supplementary Figure 1. Expression of transcription factors in freshly isolated endothelial cells from different organs with upregulation in brain endothelial cells. TF expression levels were compared between brain ECs and ECs of other vascular beds using data from three different studies (GSE47067 [4], GSE48209 [2], GSE35802 [1], GSE56777 [3, 5]). Data from a study by Nolan *et al*. (GSE47067 [4]), where brain ECs were compared to several other tissues, was analyzed using RankProdIt [6], a rank based meta-analysis tool, to generate a consensus brain EC fold-change in expression. The heatmap shows genes with the greatest fold difference. Genes were sorted based on RankProdIt fold-change (FC>1.5) in GSE47067 [4].

Supplementary Figure 2. Relative mRNA expression of EC transporter genes as compared to empty vector adenovirus control. Columns represent mean ± SD. *=P or FDR<0.05, **= P or FDR<0.01, ***= P or FDR<0.001. All experiments were performed in triplicates.

Supplementary Dataset 1. Fold-change in expression of genes in freshly isolated ECs from the BBB versus ECs from different organs as calculated using RankProdIt analysis. Raw microarray expression data from GSE47067 [4] of ECs from different vascular beds was analyzed using the Partek Genomics suite to calculate the fold-change in gene expression between ECs from brain versus other ECs of other vascular beds. RankProdIt values were calculated as a consensus summary fold-change.

Supplementary Dataset 2. List of human and mouse transcription factors as identified by the FANTOM consortium [28].

Supplementary Dataset 3. Fold-change expression of transcription factors that had a FC>1.5 in GSE47067 from studies with freshly isolated ECs from the BBB versus ECs from different organs. GSE47067 [4], GSE48209 [2], GSE35802 [1], GSE56777 [3, 5].

Supplementary Dataset 4. Evidence from literature or expression that transcription factors are relevant to induction of high-barrier resistance of ECs. The list denotes gene symbols, expression evidence from previous studies among 67 TFs that were identified from: GSE47067 [4] or literature evidence.

Supplementary Dataset 5. RNA-seq differential expression analysis at 48 h after transduction of hPSC-ECs with adenovirus overexpressing transcription factors at 80 MOI. FPKM (Fragments per Kilobase of transcript per Million mapped reads) values are listed for each gene and replicate.

Supplementary Dataset 6. RNA-seq differential expression analysis at 48 h after transduction of hPSC-ECs with adenovirus overexpressing transcription factor combinations at 20 MOI. RPKM (Reads per Kilobase of transcript per Million mapped reads) values are listed for each gene and replicate.

References:

1. Tam, S.J. *et al.* Death receptors DR6 and TROY regulate brain vascular development. *Dev. Cell* **22**, 403-417 (2012).
2. Coppiello, G. *et al.* Meox2/Tcf15 heterodimers program the heart capillary endothelium for cardiac fatty acid uptake. *Circulation* **131**, 815-826 (2015).
3. Ben-Zvi, A. *et al.* Mfsd2a is critical for the formation and function of the blood-brain barrier. *Nature* **509**, 507-511 (2014).
4. Nolan, D.J. *et al.* Molecular signatures of tissue-specific microvascular endothelial cell heterogeneity in organ maintenance and regeneration. *Dev. Cell* **26**, 204-219 (2013).
5. Daneman, R. *et al.* The mouse blood-brain barrier transcriptome: a new resource for understanding the development and function of brain endothelial cells. *PLoS One* **5**, e13741 (2010).
6. Laing, E. & Smith, C.P. RankProdIt: A web-interactive Rank Products analysis tool. *BMC Res. Notes* **3**, 221 (2010).
7. Fontijn, R.D. *et al.* SOX-18 controls endothelial-specific claudin-5 gene expression and barrier function. *Am. J. Physiol. Heart Circ. Physiol.* **294**, H891-900 (2008).
8. Keil, J.M., Liu, X. & Antonetti, D.A. Glucocorticoid induction of occludin expression and endothelial barrier requires transcription factor p54 NONO. *Invest. Ophthalmol. Vis. Sci.* **54**, 4007-4015 (2013).
9. Yuan, L. *et al.* ETS-related gene (ERG) controls endothelial cell permeability via transcriptional regulation of the claudin 5 (CLDN5) gene. *J. Biol. Chem.* **287**, 6582-6591 (2012).
10. Mishra, S., Choe, Y., Pleasure, S.J. & Siegenthaler, J.A. Cerebrovascular defects in Foxc1 mutants correlate with aberrant WNT and VEGF-A pathways downstream of retinoic acid from the meninges. *Dev. Biol.* **420**, 148-165 (2016).
11. Reyahi, A. *et al.* Foxf2 Is Required for Brain Pericyte Differentiation and Development and Maintenance of the Blood-Brain Barrier. *Dev. Cell* **34**, 19-32 (2015).
12. Birdsey, G.M. *et al.* The endothelial transcription factor ERG promotes vascular stability and growth through Wnt/beta-catenin signaling. *Dev. Cell* **32**, 82-96 (2015).
13. Matsui, T. *et al.* Redundant roles of Sox17 and Sox18 in postnatal angiogenesis in mice. *J. Cell Sci.* **119**, 3513-3526 (2006).
14. Sangwung, P., Zhou, G., Nayak, L., Chan, E.R. & Kumar, S. KLF2 and KLF4 control endothelial identity and vascular integrity. *JCI Insight* **2**, e91700 (2017).
15. Zhou, Y., Williams, J., Smallwood, P.M. & Nathans, J. Sox7, Sox17, and Sox18 Cooperatively Regulate Vascular Development in the Mouse Retina. *PLoS One* **10**, e0143650 (2015).
16. Cowan, C.E. *et al.* Kruppel-like factor-4 transcriptionally regulates VE-cadherin expression and endothelial barrier function. *Circ. Res.* **107**, 959-966 (2010).
17. Jiang, H. *et al.* Tyrosine kinase receptor B protects against coronary artery disease and promotes adult vasculature integrity by regulating Ets1-mediated VE-cadherin expression. *Arterioscler. Thromb. Vasc. Biol.* **35**, 580-588 (2015).
18. Yuan, L. *et al.* RhoJ is an endothelial cell-restricted Rho GTPase that mediates vascular morphogenesis and is regulated by the transcription factor ERG. *Blood* **118**, 1145-1153 (2011).
19. Hupe, M. & Li, M.X. Gene expression profiles of brain endothelial cells during embryonic development at bulk and single-cell levels. *Sci. Signal.* **10** (2017).
20. Deleuze, V. *et al.* TAL-1/SCL and its partners E47 and LMO2 up-regulate VE-cadherin expression in endothelial cells. *Mol. Cell. Biol.* **27**, 2687-2697 (2007).
21. Badhwar, A., Stanimirovic, D.B., Hamel, E. & Haqqani, A.S. The proteome of mouse cerebral arteries. *J. Cereb. Blood Flow Metab.* **34**, 1033-1046 (2014).

22. Costa, G. *et al.* SOX7 regulates the expression of VE-cadherin in the haemogenic endothelium at the onset of haematopoietic development. *Development* **139**, 1587-1598 (2012).
23. Liebner, S. *et al.* Wnt/beta-catenin signaling controls development of the blood-brain barrier. *J. Cell Biol.* **183**, 409-417 (2008).
24. Lim, R.G. *et al.* Huntington's Disease iPSC-Derived Brain Microvascular Endothelial Cells Reveal WNT-Mediated Angiogenic and Blood-Brain Barrier Deficits. *Cell Rep.* **19**, 1365-1377 (2017).
25. Petrovic, I., Kovacevic-Grujicic, N. & Stevanovic, M. Early growth response protein 1 acts as an activator of SOX18 promoter. *Exp. Mol. Med.* **42**, 132-142 (2010).
26. Essien, B.E. *et al.* Transcription Factor ZBP-89 Drives a Feedforward Loop of beta-Catenin Expression in Colorectal Cancer. *Cancer Res.* **76**, 6877-6887 (2016).
27. Fan, Y. *et al.* Kruppel-like factor-11, a transcription factor involved in diabetes mellitus, suppresses endothelial cell activation via the nuclear factor-kappaB signaling pathway. *Arterioscler. Thromb. Vasc. Biol.* **32**, 2981-2988 (2012).
28. Kanamori, M. *et al.* A genome-wide and nonredundant mouse transcription factor database. *Biochem. Biophys. Res. Commun.* **322**, 787-793 (2004).

Supplementary Figure S1.

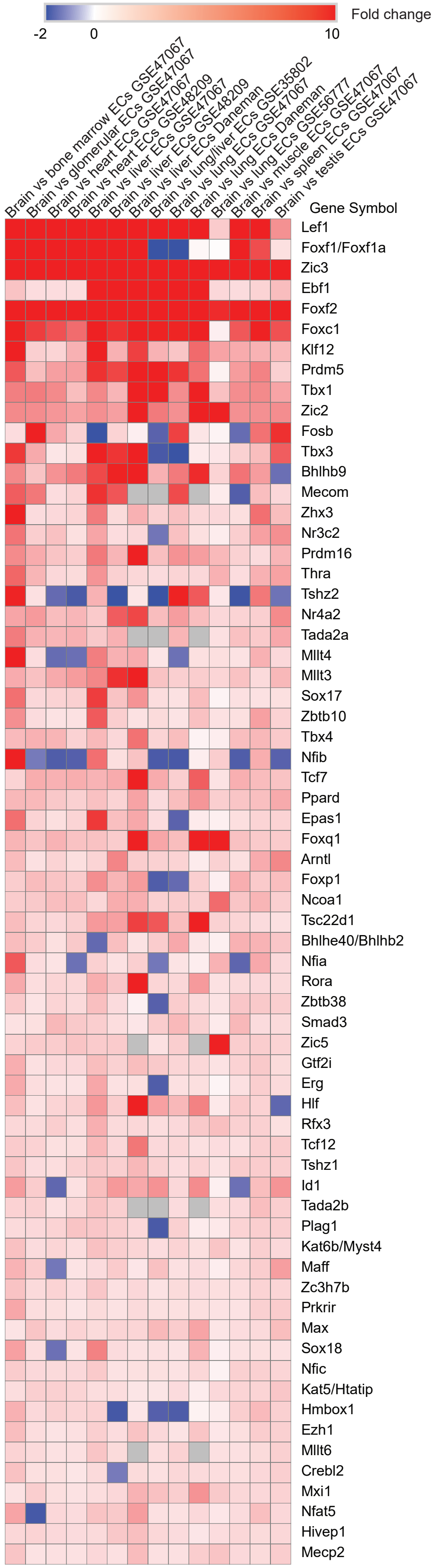

Supplementary Figure S2.

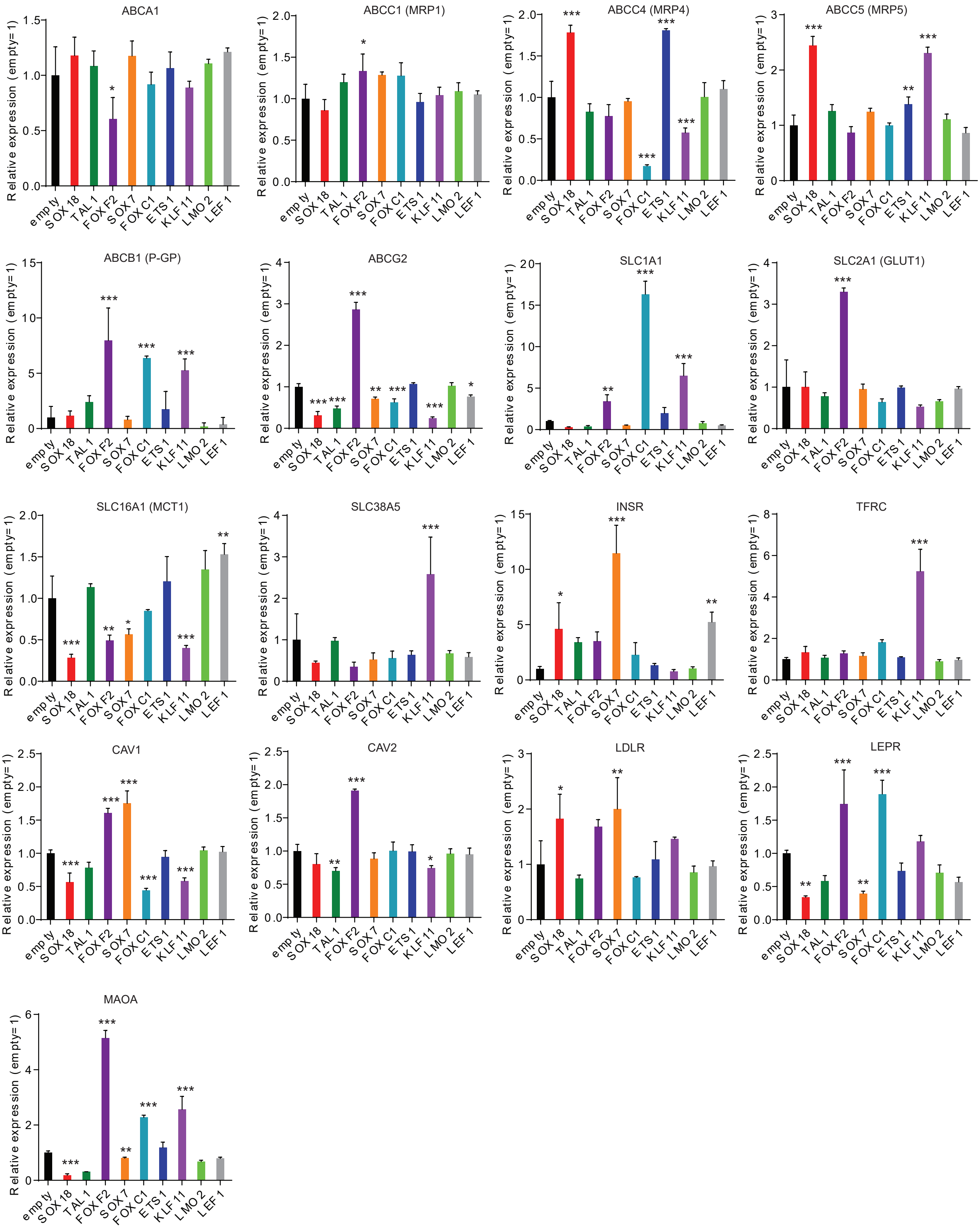

Supplement: Supplementary file 1 — Supplememtary Information. [file 41598_2020_60688_MOESM1_ESM.pdf]
